# MRI of early rectal cancer; bisacodyl micro-enema increases submucosal width, reader confidence, and tumor conspicuity

**DOI:** 10.1007/s00261-024-04701-1

**Published:** 2024-12-08

**Authors:** Ellen Viktil, Bettina Andrea Hanekamp, Arild Nesbakken, Else Marit Løberg, Ole Helmer Sjo, Anne Negård, Johann Baptist Dormagen, Anselm Schulz

**Affiliations:** 1 Department of Radiology, Oslo University Hospital Ullevål, Oslo, Norway; 2 Institution of Clinical Medicine, University of Oslo, Oslo, Norway; 3 Department of Gastrointestinal Surgery, Oslo University Hospital Ullevål, Oslo, Norway; 4 Department of Pathology, Oslo University Hospital Ullevål, Oslo, Norway; 5 Department of Radiology, Akershus University Hospital, Lørenskog, Norway

**Keywords:** Early rectal cancer, Magnetic resonance imaging, Enema, Sensitivity and specificity, Reader confidence

## Abstract

**Purpose:**

To investigate the influence of a micro-enema on diagnostic performance, submucosal width, reader confidence, and tumor conspicuity using MRI to stage early rectal cancers (ERC).

**Methods:**

In this single-center study, we consecutively included 50 participants with assumed ERC who all completed MRI with (MRin) and without (MRex) a micro-enema. The diagnostic performance was recorded for two experienced radiologists using histopathology as the gold standard. In addition, the width of the submucosa in the tumor-bearing wall, reader confidence for T-staging, and tumor conspicuity were assessed. Significance levels were calculated using McNemar’s test (diagnostic performance) and Wilcoxon’s signed-rank test (reader confidence, submucosal width, and conspicuity). Interreader agreement was assessed using kappa statistics.

**Results:**

Sensitivity/specificity were for Reader1 91%/87% for both MRex and MRin and for Reader2 74%/87% and 89%/87%, both readers p > 0.05. The micro-enema induced a significant widening of the submucosa, p < 0.001, with a mean increase of 2.2/2.8 mm measured by Reader1/Reader2. Reader confidence in T-staging and tumor conspicuity increased for both readers, p < 0.005. The proportion of tumors with both correct staging and high reader confidence increased from 58% (29/50) to 80% (40/50) (p = 0.04) for Reader1 and from 42% (21/50) to 72% (36/50) (p = 0.002) for Reader2. Interreader agreement increased from moderate (kappa 0.58) to good (kappa 0.68).

**Conclusion:**

The micro-enema significantly increased the submucosal width in the tumor-bearing wall, reader confidence, and tumor conspicuity and improved interreader agreement from moderate to good. Sensitivity and specificity in T-staging did not improve, but there was a significant increase in the proportion of tumors staged with both high confidence and correct T-stage.

**Graphical Abstract:**

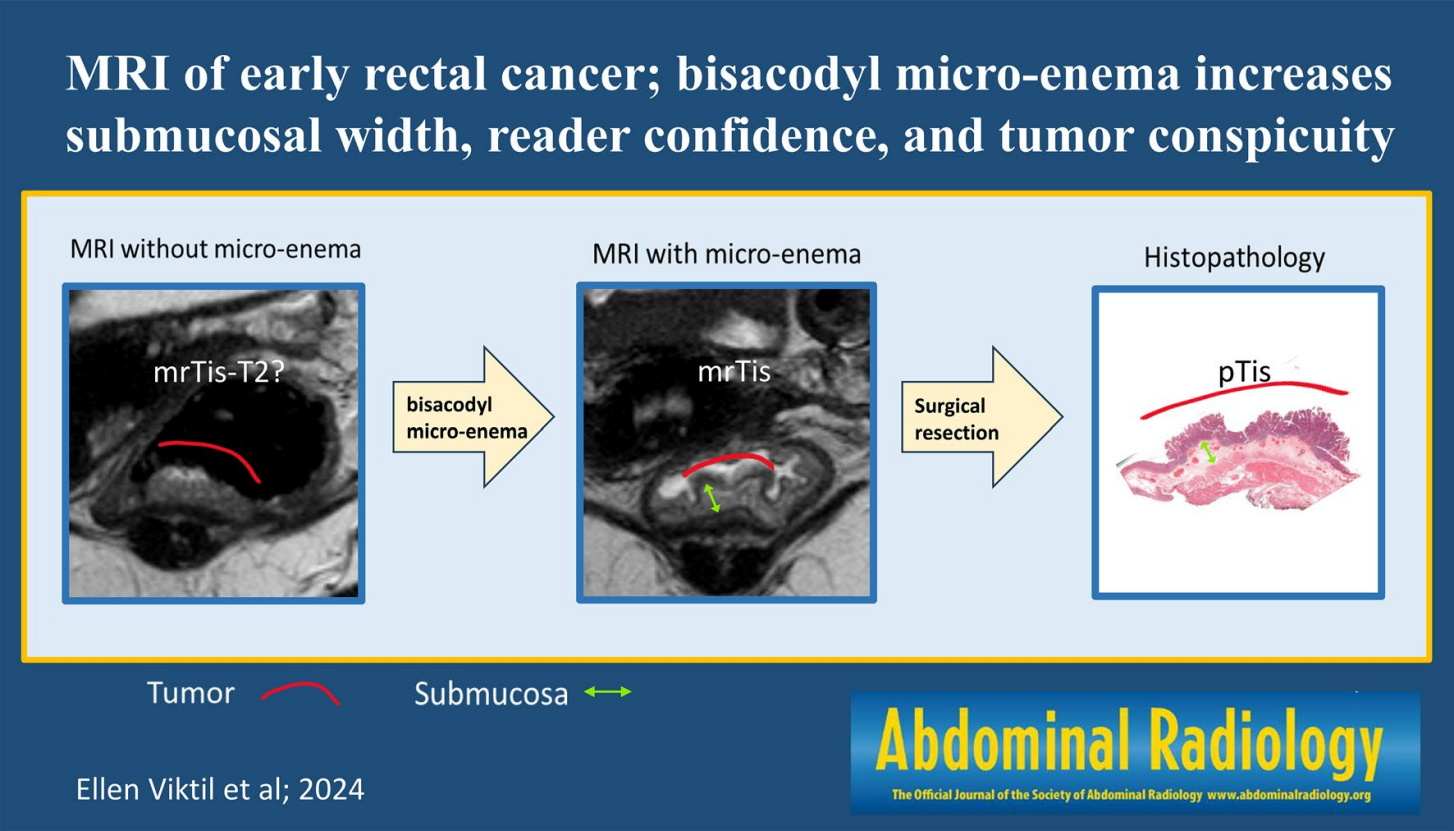

**Supplementary Information:**

The online version contains supplementary material available at 10.1007/s00261-024-04701-1.

## Introduction

Local excision (LE) of early rectal cancer (ERC) offers a definitive treatment option using organ-sparing techniques, avoiding the disadvantages of major surgery such as genito-urinary dysfunction, low anterior rectal resection syndrome, or permanent stoma after abdomino-perineal excision.

Still, early stage cancers have a risk for lymph node metastasis reported to increase with the depth of tumors submucosal invasion [1]. Newer research, however, points out high-risk factors for lymph node metastasis as poor differentiation, lymphovascular invasion, and high-grade tumor budding more important than deep submucosal invasion [2, 3]. In contrast to the high-risk factors found in histopathology after LE, the depth of submucosal tumor invasion can be determined preoperatively, and the division of submucosa into thirds, T1sm1-T1sm3, as described by Kikuchi et al. [1], is still important for the preoperative selection of candidates for LE [4]. Endorectal ultrasound (ERUS), evaluating the depth of submucosal tumor invasion, is recommended by several guidelines [4–6] but is hampered by interobserver variations [7], more recently though, the supplement of ERUS elastography has shown high interreader agreement [8]. Tumor morphology, as presented at endoscopy and stratified into the Paris classification or classification for lateral spreading tumors (LST), has been used to predict the risk for malignancy but has shown only moderate agreement among experts [9, 10]. Higher diagnostic performance has been achieved with endoscopic optical analysis using Narrow-band Imaging (NBI), evaluating micro-vessel and surface patterns as in the NICE (NBI International Colorectal Endoscopic) classification and the JNET (Japan NBI Expert Team) classification [11]. All these techniques are operator-dependent and may have limited availability.

There has been an international agreement that LE is usually curative if tumor invasion does not exceed the superficial third of the submucosa (Kikuchi T1sm1) [4, 12], while major resection is recommended for tumors invading the outer third of the (Kikuchi T1sm3) due to a 15–20% prevalence of lymph node metastases. The opinions and guidelines vary regarding the treatment of T1sm2 cancers and treatment options after LE if histopathology of the resected specimen shows high-risk factors for nodal metastasis [13].

MRI reports often do not provide sufficient information for ERC treatment decisions [14] and tend to overestimate tumor invasion in early stages, representing a severe weakness as overstaging benign or early invasive tumors can lead to unnecessary major resection or permanent stoma [15, 16]. With the increasing incidence of ERC due to the introduction of screening programs [17], it is increasingly important for radiologists to recognize benign and early-stage tumors. MRI, however, is assumed to be unable to differentiate early rectal cancers as the submucosa is often incompletely displayed on MRI [18]. Finding a method to enhance the visualization of submucosa on MRI could improve the staging of ERC.

**Fig. 1:**
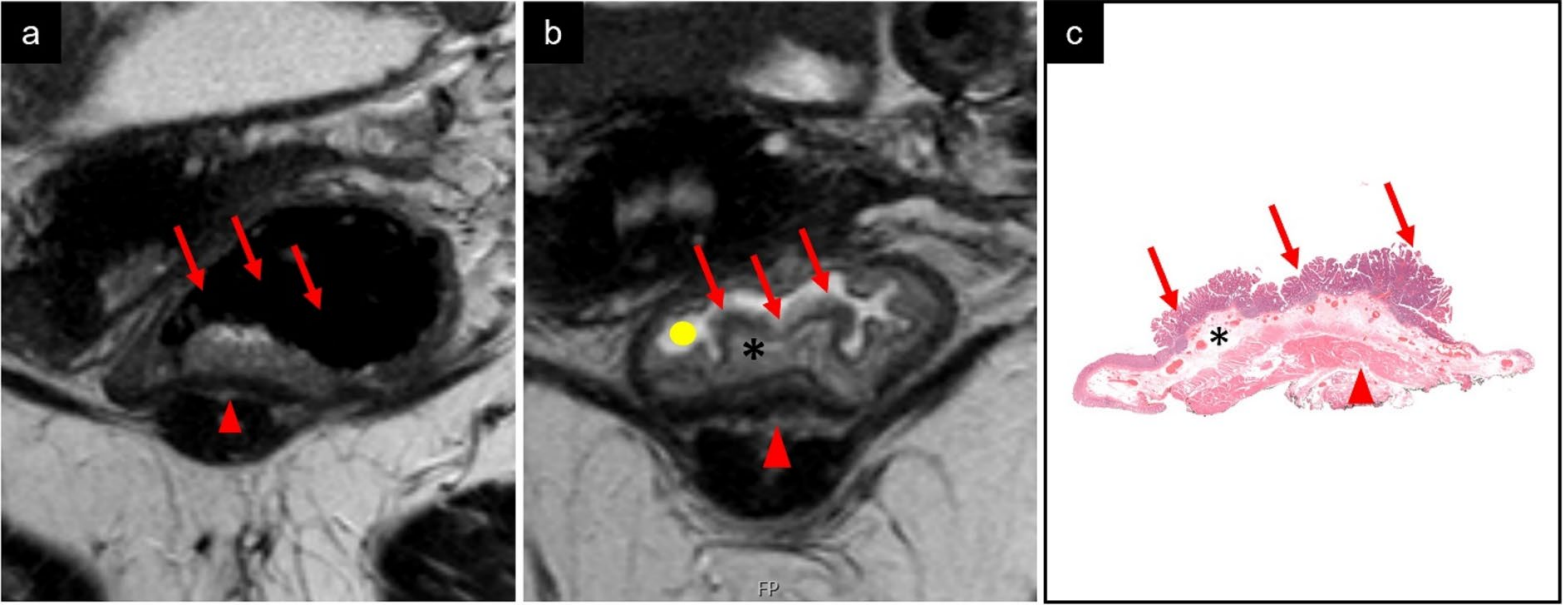
MRI, perpendicular 2D T2 TSE and histopathology of a local tumor (pTis). MR morphology; sessile polypoid. Tumor (red arrow), muscularis propria (red arrowhead), submucosa (asterix) and intraluminal fluid (yellow dot). **a** MR without micro-enema. Tumor in lower rectum. Submucosa is barely visible, and it is not possible to determine if tumor is invading submucosa. Tumor morphology is difficult to depict. **b** MR with micro-enema. Notice the widening of the submucosa and intraluminal fluid surrounding tumor. No tumor growth in submucosa visible. The sessile shape of the polyp is well displayed. **c** Histopathology showing a sessile polyp without tumor invasion into submucosa, p-Tis

Bisacodyl is a stimulating laxative approved for treating constipation and cleansing the bowel before surgical or radiological procedures [19]. We routinely apply a bisacodyl micro-enema before rectal MRI examinations and have observed that the enema generates a widening of the submucosa due to submucosal edema Fig. 1.

In this study, we wanted to investigate if a bisacodyl micro-enema can increase submucosal width in the tumor-bearing wall, improve readers' confidence and tumor conspicuity, and finally influence the diagnostic performance of MRI to identify ERC suitable for LE.

## Material and methods

The presented data in this study follow the STARD 2015 guidelines for reporting diagnostic accuracy studies [20].

### Ethics

This single-center study was approved by the local data protection officer and by the Regional Committee for Medical and Health Research Ethics South-East Norway (2015/1272). Written informed consent was obtained from all participants.

### Inclusion

**Fig. 2:**
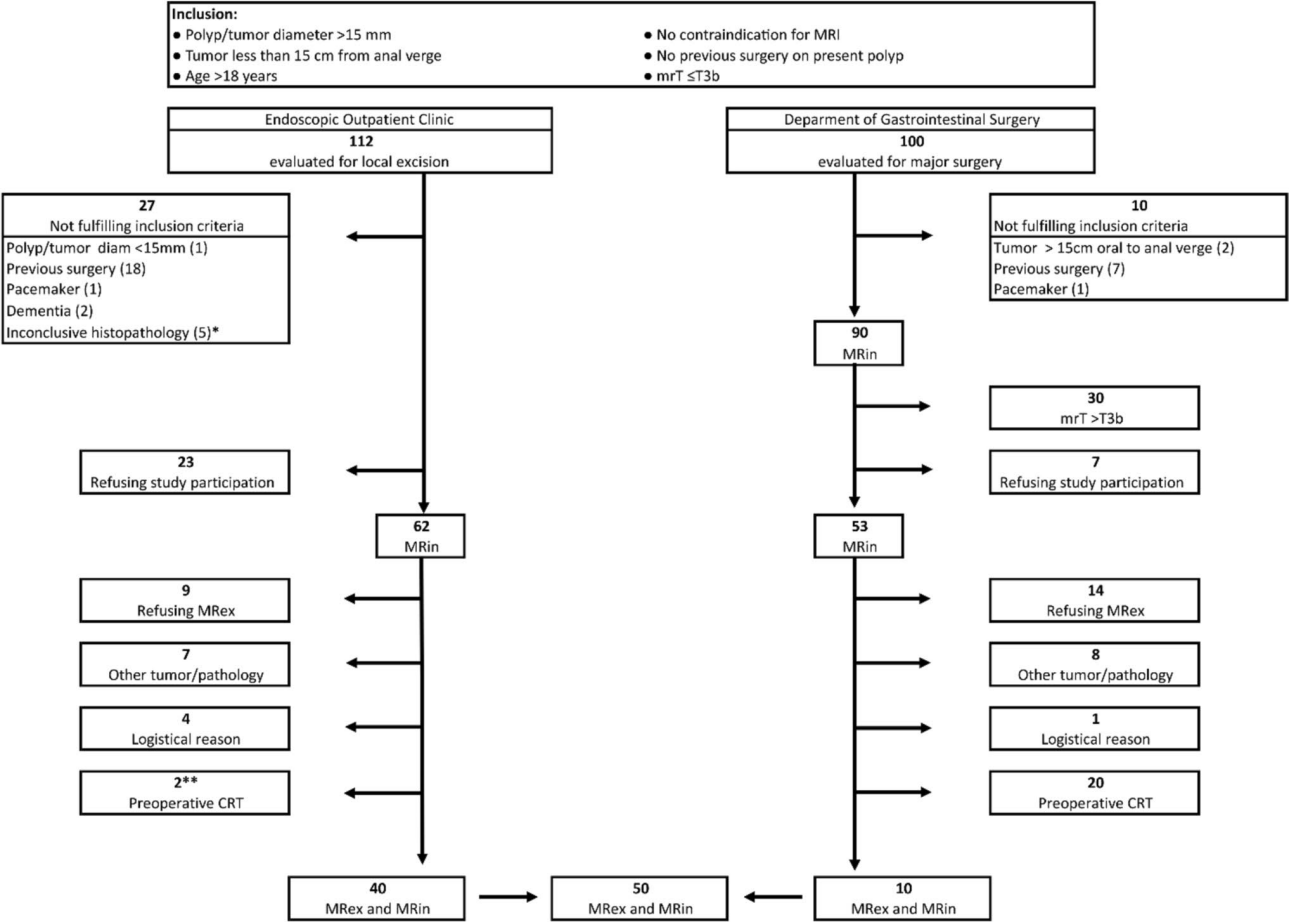
Flowchart showing inclusion and exclusion. Consecutive evaluation of all patients referred to the Endoscopic Outpatient Clinic and the Department of Gastrointestinal Surgery. mrT3b, MRI defined T-stage T3b; MRin, MR with rectal micro enema; MRex, MR without rectal micro enema; CRT, chemoradiotherapy. *2 piece meal, 1 ESD and 2 incomplete resections, **2 participants with rectal polyp and concomitant rectal cancer demanding CRT

From January 2016 to July 2017, all patients referred to our institution with newly diagnosed ERC or polyps possibly harboring ERC were evaluated for inclusion. Study participants were recruited from two different clinics, either from the Endoscopic Outpatient Clinic evaluating polyps or potentially ERC for transanal endoscopic microsurgery (TEM) using endoscopy and ERUS, or from the Department of Gastrointestinal Surgery evaluating patients with supposed or proven rectal cancer for major surgery. As most patients eligible for local excision do not routinely undergo rectal MRI, they were asked for study participation before the MRI examinations. Patients with supposed or proven rectal cancer routinely undergo an MRI of the rectum and were asked for participation if the mrT-stage was ≤ T3b. Finally, 50 participants accepted to complete two MRI examinations, one with (MRin) and one without (MRex) a bisacodyl micro-enema, Fig. 2.

Inclusion criteria were rectal polyps or tumors more than 1.5 cm in size, tumor within 15 cm of the anal verge, age more than18 years, no contraindication to perform MRI, T-stage evaluated by MRI to be ≤ T3b, and no previous surgery on present tumor/polyp. Exclusion criteria were inconclusive histopathology report for T-stage and need for preoperative chemoradiotherapy.

### MRI technique

See suppl text, suppl Table 1 and [21].

### Image analysis

Two radiologists with 20 (EV, Reader1) and 15 (BH, Reader2) years of experience in reading abdominal MRI independently interpreted the anonymized study MR-images on a multimodality reading platform (Syngovia VB30®, Siemens Healthineers). Reader1 had five years of experience reading MRI of ERC, whereas Reader2 had less experience with MRI of ERC and received an introduction before the study started. Multiplanar reformats could be viewed in any plane using the 3D T2 dataset. The readers were informed about the presence and location of a rectal tumor but otherwise blinded to all clinical information. To minimize bias from an initial learning curve and align the interpretation of study-specific variables, the readers reviewed 20 representative cases in a separate training session.

To secure at least a three-week time interval between reading MRin and MRex, each participant’s examinations were randomly assigned to two groups, each containing both MRin and MRex examinations in random order.

### Diagnostic performance

The T-stage of each tumor was assigned to a 15-point scale derived from the UICC TNM 8 classification system [22] and expanded with subclasses for T1 (sm1, sm2, and sm3), T2 (inner and outer muscular layer), and T3 (T3a – T3d). To compare the diagnostic performance of MRin and MRex, the tumors were stratified into two groups, “local” tumors (Tis—T1sm2) and appropriate for LE, and all more advanced tumors (T1sm3 – T3b) to the group “non-local” and too invasive for LE [21]. Registration on a 15-point scale also allowed for calculating diagnostic performance using the UICC TNM8 system.

### Bowel wall

Both readers registered the minimum and maximum width of submucosa in the tumor-bearing bowel wall on both MRin and MRex. Reader1, in addition, measured the sagittal rectal width at tumor height.

### Reader’s confidence

Reader's confidence in diagnostic performance was assessed using an 11-point figurative visual analog scale (fVAS) with reference points at 0, 3.3, 6.6, and 10 cm [23]. Confidence in T-staging was scored from 0 = not confident to 10 = highly confident, and the confidence levels were recorded with a 10 cm ruler, Suppl Fig. 1.

To evaluate the effect of the micro-enema on the reader's confidence for T-staging, the proportions of tumors staged correctly with high confidence (score ≥ 6.6) were determined.

To ensure that the effect of the micro-enema was not purely an increase in readers' confidence in general, incorrect staging was given a negative weighting by multiplying the readers' confidence score with -1, as proposed by Tsushima et al. [24]. The new variable was called “the true confidence”.

### Tumor conspicuity

Tumor conspicuity was defined by two visual qualitative features: delineation of tumor base and tumor discrimination from bowel contents. It had predefined image criteria and was scored from 0 = bad to 10 = excellent (Suppl Fig. 2).

To register the amount of luminal peritumoral fluid, Reader1 recorded if the tumor was more than 50% surrounded by fluid.

### Tumor morphology

As there is neither an MRI specific classification for the morphology of rectal polyps nor an MRI based system corresponding to the Paris classification, the readers registered the tumors as semiannular, non-polypoid flat, sessile lobulated, sessile polypoid, pedunculated, or complex polypoid, a classification system based on morphology as displayed on MRI and exemplified to the readers as figures and images.

### Intra and interreader agreement

Kappa values for intra- and interreader agreement for diagnostic performance were calculated. For tumor conspicuity and morphology interreader kappa values were calculated.

Kappa values were interpreted according to Altman: κ < 0.20 as poor, κ of 0.21–0.40 as fair, κ of 0.41- 0.60 as moderate, κ of 0.61- 0.80 as good, and κ of 0.81–1.00 as excellent agreement.

### Reference standard

All participants underwent surgical resection and histopathological examination of the resected specimen was used as the reference standard for T-staging. The histopathology sections were stained with hematoxylin and eosin (HE) and evaluated by a pathologist with more than 30 years of experience in colorectal tumors.

### Statistical analyses

All statistical analyses were performed using Stata® (Statistical Software: Release 16. College Station, TX: StataCorp LLC). The diagnostic performance for T-staging in terms of sensitivity, specificity, positive predictive value (PPV), negative predictive value (NPP), and area under the receiver operating characteristic curve (ROC AUC) was calculated using the Stata module Diagt [25]. The significance level for diagnostic performance for T-stage and proportions of tumors staged correctly with high confidence was calculated with McNemar’s test.

T-test was used for normally distributed data, and the Wilcoxon signed-rank test for non-normally distributed data. For intra- and interreader agreements, Cohen’s Kappa was calculated. P-values of ≤ 0.05 were considered significant.

To achieve 80% statistical power to detect a significant difference in diagnostic performance between MRex and MRin, assuming 60% pretest correctness for MRex and 80% for MRin, we estimated that 55 participants should be included.

## Results

In total, 50 participants underwent both MRin and MRex and were included in the study. The median time interval between the examinations was 18 (2–41) days. Except for one participant, the time interval between the interpretation of MRex and MRin was at least five weeks.

In total, 70% (35/50) were local tumors, of which 89% (31/35) were Tis and 11% (4/35) were T1sm1-sm2. Participant and tumor characteristics are shown in Table 1.

**Table 1:** Participant and tumor characteristics

Variable	n (%) // median (range)
Age	65 (46–90)
Sex	
Men	26
Women	24
Tumour size:	
Tumor length local, mm	35 (15—120)
Tumour length non-local, mm	42 (20—68)
Location tumour:	
Low rectum, 0–5 cm from anal verge	17 (34)
Midrectum, > 5–10 cm from anal verge	28 (56)
Upper rectum, > 10–15 cm from anal verge	5 (10)
Histopathology, benign vs malignant	
Benign (Tis)	31 (62)
Malign (> Tis)	19 (38)
Histopathology, local vs non-local	
Local (Tis- T1sm2)	35 (70)
Non-local (T1sm3-T3b)	15 (30)
Histopathology with subclassifications	
Tis	31 (62)
T1sm1	3 (6)
T1sm2	1 (2)
T1sm3	3 (3)
T2 inner	6 (12)
T2 outer	3 (6)
T3a	0 (0)
T3b	2 (4)
T3c	1 (2)

*Tis* tumor in situ; *sm1, sm2, and sm3* submucosal staging after Kikuchi

### Diagnostic performance

When comparing the diagnostic performance for T-staging, we found a slight improvement in applying the micro-enema for one reader only, but statistical significance was not reached (p = 0.18) Tables 2 and 3. For the results using the UICC TNM8 system, see suppl Tables 2 and 3.

**Table 2:** Diagnostic performance for T-stage for Reader1 and Reader2 without (MRex) and with (MRin) a rectal micro-enema

	sens (CI), n-cor/n-local	spec (CI), n-cor/n-nonlocal	PPV (CI)	NPV (CI)	roc (CI)	N (total)
Reader1						
Local MRex	91 (77- 98) 32/35	87 (60–98) 13/15	94 (80–99)	81 (54–96)	0.89 (0.79–0.99)	50
Local MRin	91 (77- 98) 32/35	87 (60–98) 13/15	94 (80–99)	81 (54–96)	0.89 (0.79–0.99)	50
Reader2						
Local MRex	74 (57- 88) 26/35	87 (60–98) 13/15	93 (77–99)	59 (36–79)	0.8 (0.69–0.92)	50
Local MRin	89 (73- 99) 31/35	87 (60–98) 13/15	94 (78–99)	77 (50–93)	0.88 (0.77–0.98)	50
Reader1						
Non-local MRex	87 (60–98) 13/15	91 (77- 98) 32/35	81 (54–96)	94 (80–99)	0.89 (0.79–0.99)	50
Non-local MRin	87 (60–98) 13/15	91 (77- 98) 32/35	81 (54–96)	94 (80–99)	0.89 (0.79–0.99)	50
Reader2						
Non-local MRex	87 (60–98) 13/15	74 (57- 88) 26/35	59 (36–79)	93 (77–99)	0.8 (0.69–0.92)	50
Non-local MRin	87 (60–98) 13/15	89 (73- 97) 31/35	77 (50–93)	94 (80–99)	0.88 (0.77–0.98)	50

*Local* Tis -T1sm2, *non-local* T1sm3-T3b, *sens* sensitivity, *spec* specificity, *PPV* positive predictive value, *NPV* negative predictive value, *CI* confidence interval, *n_cor* number correct, *n-local* total number local tumors, *n-nonlocal* total number non-local tumors, *n-total* total number participants

**Table 3:** Contingency table comparing the diagnostic performance for R1 and R2 with histopathology using the modified classification system, without (MRex) and with (MRin) a micro-enema

	Tis	sm1	sm2	sm3	T2in	T2out	T3a	T3b	T3c	Total
Reader1 MRex										
Local	31	1	0	2	0	0	0	0	1	35
Non-local	2	0	0	2	1	1	3	6	0	15
Total	33	1	0	4	1	1	3	6	1	50
Reader1 MRin										
Local	28	1	3	3	0	0	0	0	0	35
Non-local	2	0	0	0	3	2	2	6	0	15
Total	30	1	3	3	3	2	2	6	0	50
Reader2 MRex										
Local	15	8	3	3	4	1	1	0	0	35
Non-local	2	0	0	0	3	3	3	4	0	15
Total	17	8	3	3	6	4	4	4	0	50
Reader2 MRin										
Local	22	5	4	3	1	0	0	0	0	35
Non-local	2	0	0	3	3	0	4	3	0	15
Total	24	5	4	6	4	0	4	3	0	50

*Local* Tis -T1sm2**,** *non-local* T1sm3-T3b, *Tis* tumor in situ, *sm1-sm3* grading of submucosal tumor invasion after Kikuchi =

Reader1 overstaged 9% (3/35) of the tumors on MRin and MRex. Reader2 overstaged 26% (9/35) of the tumors on MRex and 11% (4/35) on MRin. Both readers understaged 13% (2/15) on both MRex and MRin

### Bowel wall

**Fig. 3:**
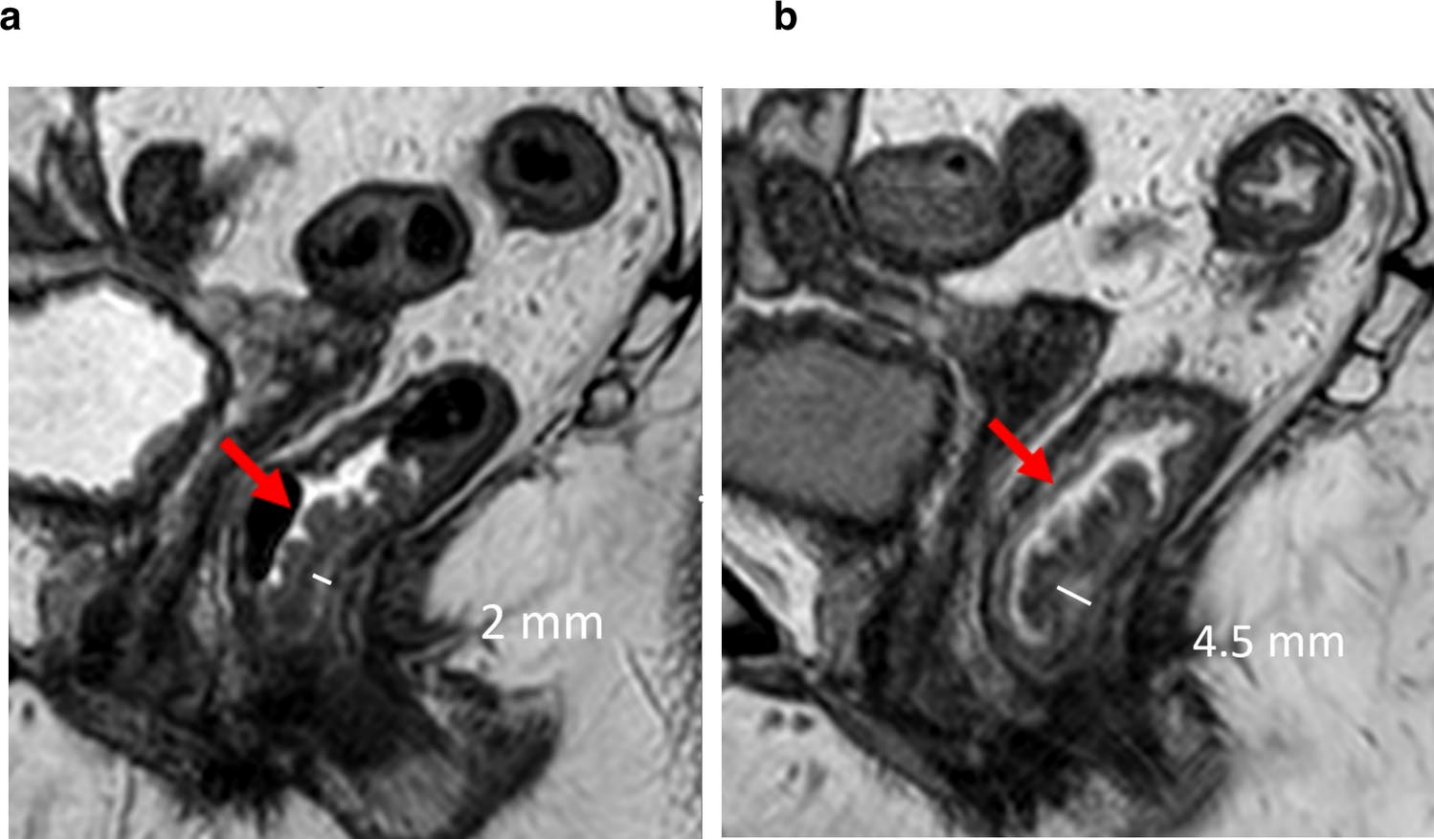
Sagittal 3D T2 of a local tumor (pTis) tumor (red arrow) submucosa (white line). MR morphology; sessile lobulated. **a** Without micro-enema. Tumor in lower rectum, submucosal width 2mm in tumor bearing wall. Tumor partly surrounded by mucus. **b** After micro-enema. Widening of submucosal width to 4.5 mm in tumor bearing wall. Tumor entirely surrounded by mucus and fluid

The micro-enema induced a significant widening of the submucosa in the tumor-bearing wall, see Fig. 3 and Table 4.

**Table 4:** Minimum and maximum width of submucosa in the tumor-bearing wall measured by Reader1 and Reader2

	MRex mean/median (min–max) mm	MRin mean/median (min–max) mm	p-value
Reader1 minimum width	0.1/0 (0–2)	0.7/2 (0–6)	0.02
Reader1 maximum width	1.7/2 (0–5)	3.8/3 (0–17)	< 0.001
Reader2 minimum width	0.1/0 (0–2)	1.2/1 (0–4)	< 0.001
Reader2 maximum width	0.8/1 (0–4)	3.6/4 (0–10)	< 0.001

*MRex* MR without micro-enema, *MRin* MR with micro-enema

Reader1 measured a mean increase ± standard deviation of submucosal width of 2.2 ± 2.9 mm and Reader2 of 2.8 ± 2.3 mm. The mean rectal width ± standard deviation at tumor level did not change, measuring 29.0 ± 10.3 mm on MRex and 29.4 ± 7.9 mm on MRin at tumor height.

### Reader’s confidence

**Fig. 4:**
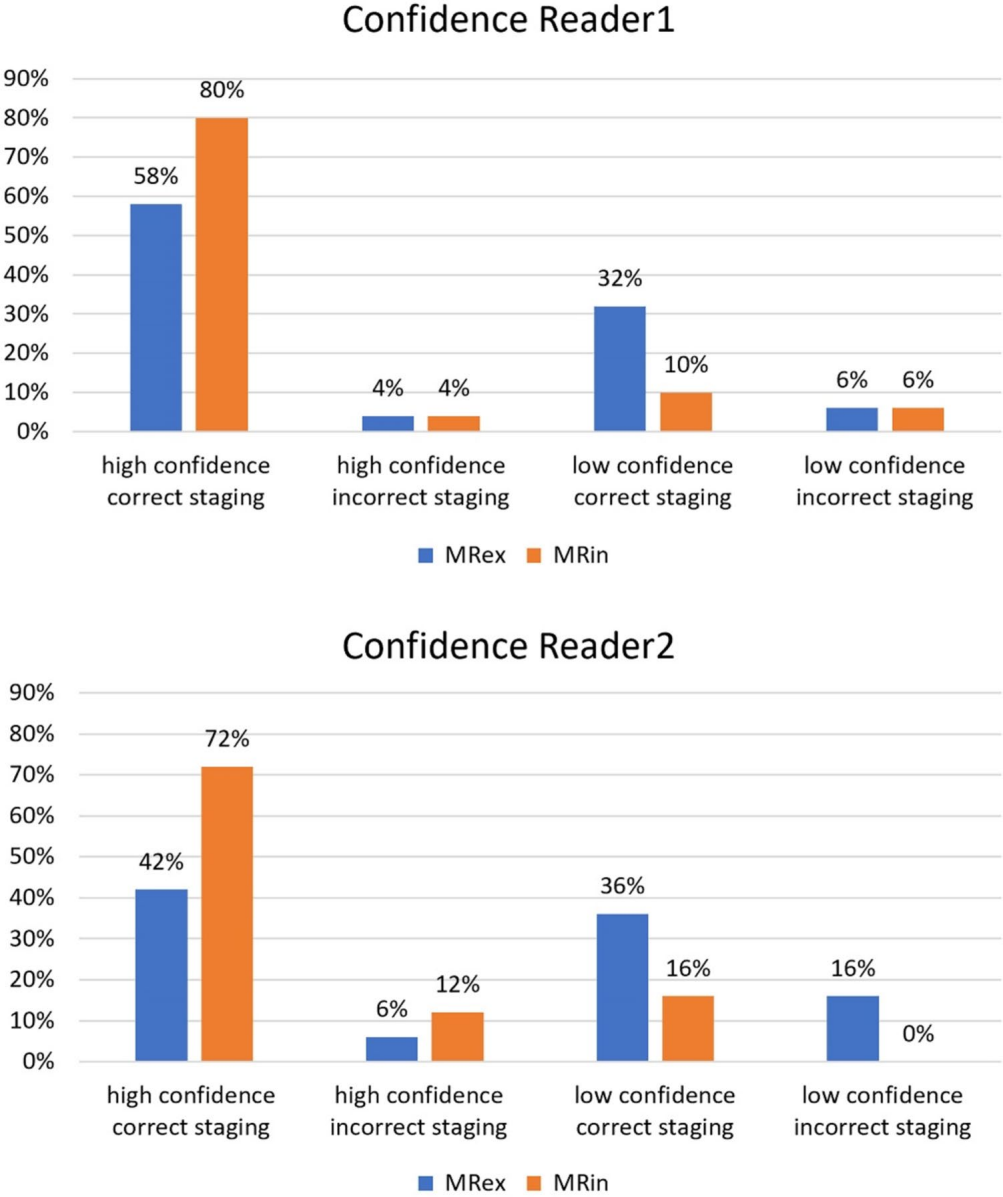
Reader’s confidence, high or low, combined with correct or incorrect staging of the tumors as local or non-local

There was a significant increase in the readers' confidence in T-staging and depiction of tumor morphology after applying a micro-enema, Table 5. In addition, the micro-enema increased the proportion of tumors staged correctly with high confidence for both readers, for Reader1 from 58% (29/50) on MRex to 80% (40/50) on MRin (p = 0.04) and for Reader2 from 42% (21/50) on MRex to 72% (36/50) on MRin (p = 0.002), Fig. 4.

**Table 5:** Median values ± standard deviation for readers' confidence, readers' true confidence, tumor conspicuity and morphology. Tumor conspicuity is assessing the ability to discriminate the tumor from bowel contents and to delineate the tumor base

	MRex	MRin	p-value
Reader1			
Confidence T-stage	6.6 ± 2.5	10 ± 2.2	0.005
True confidence T-stage	6.6 ± 4.7	10 ± 4.7	0.006
Discrimination tumor—bowel contents	7.8 ± 3.0	10 ± 1.8	< 0.001
Delineation tumor base	6.6 ± 2.7	9.5 ± 2.1	< 0.001
Confidence tumor morphology	6.6 ± 3.6	10 ± 1.9	< 0.001
Reader2			
Confidence T-stage	6.5 ± 2.0	8.5 ± 2.1	< 0.001
True confidence T-stage	6.2 ± 4.6	8.5 ± 5.2	0.006
Discrimination tumor—bowel contents	6.0 ± 2.1	9.0 ± 2.5	< 0.001
Delineation tumor base	4.0 ± 2.3	8.5 ± 2.5	< 0.001
Confidence tumor morphology	6.2 ± 1.8	7.2 ± 2.0	< 0.001

*MRex* MR without micro-enema, *MRin* MR with micro-enema

Both readers had some cases of incorrect staging with high confidence; for Reader1, this remained unchanged at 4% (2/50) on MRex and MRin but increased from 6% (3/50) at MRex to 12% (6/50) at MRin for Reader2, Fig. 4. For the true reader confidence, the micro-enema still led to a significant increase in median confidence levels, Table 5.

### Tumor conspicuity

**Fig. 5:**
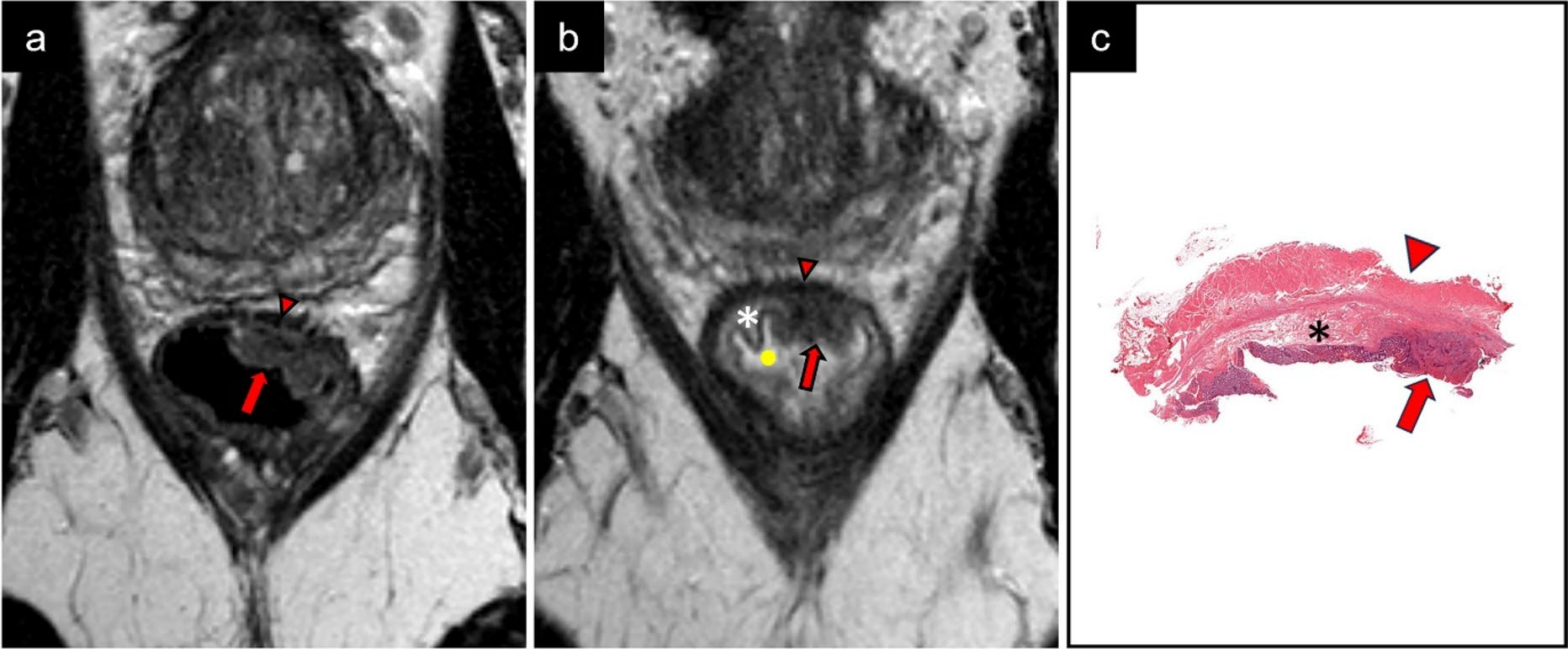
MRI and histopathology of a non-local tumor, pT1sm3. MRI morphology; semiannular. Perpendicular MR images (2D T2 TSE). Tumor (red arrow), muscularis propria (red arrowhead), submucosa (white/black asterix) and intraluminal fluid (yellow dot). **a** MRI without micro-enema. Tumor in lower rectum. Submucosa is barely visible, and it is not possible to determine the depth of tumor invasion or define tumor morphology. **b** MRI with micro-enema. Widening of submucosa and tumor growth in submucosa visible. Increasing amount of luminal fluid. **c** Histopathology showing pT1sm3

There was a significant improvement in tumor conspicuity, both in tumor delineation and discrimination from bowel contents, see Table 5. The number of tumors more than 50% surrounded by fluid increased from 37% (18/49) on MRex to 90% (45/50) on MRin Figs. 1, 3, and 5.

### Tumor morphology

Both readers classified most tumors as semiannular, sessile polypoid, or sessile lobulated. When collapsing the sessile polyps to one class using the results from MRin displaying the morphology most clearly, Reader1 registered 28% (14/50) as semiannular, 58% (29/50) as sessile, leaving a rest group of miscellaneous morphologies of 14% (14/50). Reader2 recorded 34% (17/50) as semiannular, 48% (24/50) as sessile, and a rest group of 18% (9/50). Reader1 recorded four pedunculated polyps in the rest group, and Reader2 one.

When correlating the reader's classification of tumor morphology to histology, 73% (11/15) of the tumors rated as semiannular on both MRex and MRin by Reader1 were non-local at histopathology, whereas 87% (13/15) rated as semiannular on MRex and 67% (10/15) on MRin by Reader2, were non-local. Regarding the polyps recorded as sessile by Reader1, 86% (30/35) on MRex and 91% (32/35) on MRin were local at histopathology. For Reader2, 40% (14/35) on MRex and 80% (28/35) on MRin were local at histopathology.

### Intra and interreader agreement

For intra-reader agreement for T-stage comparing MRex with MRin, Reader1 achieved a kappa value of 0.72 and Reader2 0.63. Regarding interreader agreement for the T-stage, the readers achieved kappa values of 0.58 on MRex and 0.68 on MRin. For tumor conspicuity the kappa values raised from MRex to MRin from 0.34 to 0.61 for delineation of tumor base, and from 0.27 to 0.47 for discrimination of tumor from bowel contents. For tumor morphology the kappa value for interreader agreement raised from 0.22 on MRex to 0.58 on MRin.

## Discussion

In this study, we used bisacodyl micro-enema as a preparation before MRI of ERC. The micro-enema led to a significant widening of the submucosa in the tumor-bearing wall, increased readers' confidence and interreader agreement in T-staging, tumor conspicuity and morphology. Finally, this resulted in a slight but insignificant improvement in diagnostic performance for the reader with less experience in MRI of ERC. However, when combining diagnostic performance with reader confidence, the proportions of tumors staged with both high confidence and correct T-stage increased significantly for both readers.

The use of rectal filling with fluid or gas to enhance tumor visualization and staging has been investigated by several authors [26–29]. There has been a concern that using rectal filling media could lead to distension of the rectum and reduced distance between the tumor and mesorectal fascia (MRF), thereby causing unnecessary preoperative therapy [27]. However, neither glycerin enema nor gel was found to reduce the distance between the tumors and MRF [28, 29]. Rectal filling with water or glycerin enema improved the visualization and T-staging of rectal tumors [26, 28], whereas the use of gel tended to overstaging, especially in early tumors [29]. These partly conflicting results may be related to differences in the medium and volume used for rectal filling.

More recently, rectal micro-enemas have been advocated in the response evaluation after chemoradiotherapy (CRT) [30, 31] as they remove bowel gas and decrease susceptibility artifacts, thereby improving diffusion-weighted imaging (DWI). Still, there is no consensus on using pre-procedure preparations as micro-enemas or rectal filling, and they are not routinely advised by the ESGAR recommendation [5].

Bisacodyl micro-enema, used in this study, is a prodrug metabolized in the intestinal mucosa and has a dual action by stimulating bowel motility and fluid secretion [19]. We report on a third effect, the induction of submucosal edema, a finding already registered by Ciggaar et al. investigating MRI of endometriosis [32].

In our study, the fluid secretion induced by the micro-enema significantly increased the number of tumors surrounded by fluid, enhancing tumor conspicuity. In line with Coskun et al. [33], we found no increased distension of the rectum at tumor height, diminishing the risk for compression of the mesorectum and, subsequently, overtreatment of tumors due to the shortening of the distance to MRF.

The main aim of the study was to evaluate if the widening of the submucosa could improve the T-staging of ERC by better displaying the layers of the rectal wall and improving the depiction of the tumor's invasive front. Although both readers measured a significant increase in the width of the submucosa in the tumor-bearing bowel wall on MRin, there was no improvement in the diagnostic performance for Reader1. Reader2 achieved a slight but statistically insignificant improvement. Nevertheless, for Reader2, this may imply a clinical effect as the number of correct staged tumors increased from 78% (39/50) to 88% (44/50), and a significant increase in diagnostic performance could potentially have been achieved with a larger study selection. In addition, the interreader agreement for T-stage, tumor conspicuity and tumor morphology improved on MRin, indicating better visualization of the tumor and factors influencing T-staging.

MRI is usually not performed on benign to early stage tumors, as it is hampered by overstaging [4, 15]. Overtreatment, though, is still a problem [16, 34] and as screening is bringing a shift to earlier stages, it is essential to raise the focus on early stages and MRI, as some of these patients probably will undergo an MRI examination. To be able to record the effect of a micro-enema on overstaging, we aimed for a study population containing a specter of tumors from benign and early stages to invasive cancers ≤ mrT3b. Achieving the optimal study population is demanding, and to obviate the small clear-cut benign tumors, we only included polyps larger than 15 mm. Finally, the consecutive inclusion resulted in a slight overweight of benign tumors.

The diagnostic performance and rate of misclassification achieved by the readers on MRin were in line with comparable ERUS studies investigating similar T-stage selections, with sensitivities ranging from 71–95%, specificities 62–100%, and overstaging 5–27% [35–40]. Misclassification of ERC is problematic, especially for overstaging, as overstaging can lead to overtreatment and an unnecessary rectal amputation. Both readers overstaged some tumors; however, for Reader2, the micro-enema reduced the number of overstaged tumors from nine to four on MRin. The number of understaged tumors was low for both readers and was not influenced by the micro-enema.

One possible reason for the micro-enema's low impact on overall diagnostic performance could be the evaluation method used. The readers were asked to select a T-stage without an alternative for indeterminate lesions and their uncertainty regarding T-stage, may not have been depicted.

Another reason for the absence of a significant improvement in diagnostic performance may be that both readers were experienced in interpreting MRIs of locally advanced rectal cancers, as the use of inexperienced readers may better demonstrate the effect of new technologies [41]. This applies especially to Reader1, who was also experienced in ERC and had no effect of the micro-enema on diagnostic performance in the study.

The reader's confidence was measured separately, and the micro-enema significantly increased the reader's true confidence for T-staging, and the proportions of tumors staged correctly with high confidence increased for both readers. In addition, the readers confidence for tumor conspicuity and tumor morphology raised from fair to moderate or good. Reader's confidence is essential as it can impact several factors, such as reading time, clarity of reports, and diagnostic performance [42]. Clinicians may also be more inclined to act on radiology reports expressed with higher confidence [43]. On the other hand, several conditions may influence readers' confidence, such as experience, image quality, clinical information, and CAD- assistance, and several studies have shown improvement in reader confidence by refinement of imaging techniques [44–47]. As a reader's confidence is crucial in communicating radiology reports, a higher confidence achieved after a bisacodyl micro-enema may improve the quality of the reports and increase the impact on clinical practice.

We found a connection between specific tumor morphologies and tumor histology, with semiannular morphology indicating non-local tumors and sessile morphology local tumors. This suggests that tumor morphology, as shown on MRI, could play a role in tumor staging. Furthermore, the micro-enema increased the number of tumors with sessile morphology proven as local at histopathology, especially for Reader2, meaning that morphology after a micro-enema potentially can contribute to less overstaging.

The readers noted some tumors as pedunculated, but considering the stalks being only 3–6 mm long, measured on MRin in retrospect, the polyps rather qualify as subpedunculated, which may explain the lack of agreement between the readers.

A strength of the study is the use of histopathology as the gold standard and the prospective inclusion with paired participant and reader design. This reduces the number of confounding factors and raises the level of evidence for comparison.

In this study, we investigated the reader's confidence in T-staging and visual evaluation of tumor conspicuity, which are all subjective parameters difficult to reproduce among readers. To minimize disagreement, we tried to align the Reader's evaluation by defining criteria for visual scoring and using an fVAS with absolute minimum and maximum scores and two reference points in between.

Our study has some limitations. Twenty-three participants refused MRex, which may have led to a bias, but as we believe the reasons were random, we consider the bias minimal. Further, we did not achieve the required sample size, as only 50 participants admitted undergoing a second MRI examination and we included two readers only, a number lower than recommended for multiple-reader studies [41]. Both conditions may limit the statistical power to detect clinically significant differences.

Furthermore, because of the apparent signs of a rectal micro-enema, such as a clean bowel, submucosal edema, and fluid in the lumen, it was impossible to hide the exposure to an enema, and the study cannot be regarded as truly blinded. However, we tried to minimize the reader's recall bias by assigning MRex and MRin to two reading sessions separated by a minimum interval of four weeks. In addition, within each reading session, MRin and MRex were presented in a random order.

Moreover, as both readers were experienced readers of MRI of rectal cancer, the study's outcome cannot be generalized to the overall population of radiologists but merely to radiologists working in specialist centers in close cooperation with referring clinicians and pathologists.

As bisacodyl micro-enema enhances the layers of the bowel wall, the method may help image various conditions affecting the rectum. Further research is needed to explore its potential in imaging endometriosis to assess the possibility of easier identification and grading of deep infiltration. In combination with DWI, the method may also be advantageous in response evaluation of rectal cancer after CRT to help identify complete responders or in follow-up for early detection of regrowth or detection of recurrency after LE of rectal tumors. The improved display of polyp morphology could facilitate the development of an MRI classification system for polyps and ERC with possible correlation to clinical classifications and histopathology.

In conclusion, the supplement of a rectal micro-enema induced a significant increase in the submucosal width in the tumor-bearing wall. The micro-enema also led to higher reader confidence and interreader agreement for diagnostic performance, tumor conspicuity and morphology, and tumor morphology as depicted on MRI may contribute to correct T-staging. Diagnostic performance alone was not improved; however, the micro-enema significantly raised the proportion of tumors correctly staged with high confidence, which in a clinical setting may lead to more precise reports and a greater impact on clinical practice.

## Supplementary Information

Below is the link to the electronic supplementary material.

Supplementary file1 (DOCX 159 KB)

## Data Availability

No datasets were generated or analysed during the current study.
