# Supplementary material for: MRI of early rectal cancer; bisacodyl micro-enema increases submucosal width, reader confidence, and tumor conspicuity
Source: Abdom Radiol (NY). 2024 Dec 8;50(6):2401–13. doi: 10.1007/s00261-024-04701-1 (PMC12069511; doi:10.1007/s00261-024-04701-1)
Supplement: Supplementary file 1 — Supplementary file1 (DOCX 159 KB) [file 261_2024_4701_MOESM1_ESM.docx]

Supplementary Information SI

Supplementary text: MRI technique

For bowel cleansing, a micro-enema (5 ml bisacodyl 10 mg/ml; Toilax®, Orion) was administered one hour before the examination. If not contraindicated, all participants received 1 mg glucagon (Glucagon®, Novo Nordisk Denmark) i.m prior to the examination and 20 – 40 mg of butylscopolamine (Buscopan®, Sanofi-Aventis UK) i.v during the examination to reduce bowel motion artifacts.

The MRI examinations were performed on a Philips Achieva 1.5 T (Philips Medical Systems, Einthoven, The Netherlands) with a 32-channel cardiac coil or a Siemens Aera 1.5 T (Siemens Healthineers, Erlangen, Germany) with a body 30 coil. From the promontory to the anal verge, an axial 3D T2 weighted sequence (T2W) with sagittal and coronal reformats was acquired. The sequence was close to isotropic and allowed post-processing of multiplanar reformats in any direction. At tumor height, two high-resolution 2D T2W turbo spin-echo images were acquired, one perpendicular and one parallel to the long axis of the rectum and tumor. Diffusion-weighted imaging was performed in two plans, one axial full field of view from the promontory to the anal verge and one high-resolution angulated parallel to the long tumor axis, identical to the angulation plane of the high-resolution 2D T2W turbo spin-echo. The detailed acquisition parameters are provided in Suppl Table 1.

Table SI 1 MR imaging acquisition parameters for Philips Achieva 1.5 T and Siemens Aera 1.5T

| **1.5 Tesla (Phillips Achiva)** | **T2W TSE** | **T2W TSE** | **T2 3D VISTA**^a^ | **DWI** | **DWI** | **T1 3D VISTA** |
| --- | --- | --- | --- | --- | --- | --- |
| Imaging planes | Oblique axial^b^ | Oblique parallel^c^ | Axial with sagittal and coronal reformates | Oblique parallel | Axial | Coronal |
| Repetition time/Echo time(msec) | 4695/90 | 4695/90 | 1175/85 | 1262/83 | 2808/69 | 250/14 |
| Bandwidth (Hz/pixel) | 195 | 195 | 445 | 2234 | 2629 | 430 |
| Field of view (mm) | 160 x 160 | 160 x 160 | 289/190 | 20 x 20 | 330 x 330 | 460 x 460 |
| Acquisition Matrix | 256/224 | 256/224 | 292/252 | 80 x 117 | 112 x 107 | 3841/382 |
| Section thickness/gap (mm) | 3/0.3 | 3/0.3 | 1/-0.5 | 5/1 | 5/1 | 1.2/-0.6 |
| Number of signals acquired/b-value | 6 | 6 | 1 | 8/b-0  8/b-500  8/b-1000 | 3/b-0  3/b-500  3/b-1000 | 2 |

| **1.5 Tesla (Siemens Aera)** | **T2W TSE** | **T2W TSE** | **T2 3D SPACE**^d^ | **DWI** | **DWI** | **T1 3D SPACE** |
| --- | --- | --- | --- | --- | --- | --- |
| Imaging planes | Oblique axial | Oblique parallel | Axial with sagittal and coronal reformates | Oblique parallel | Axial | Coronal |
| Repetition time/Echo time(msec) | 4250/82 | 4250/82 | 1300/90 | 3400/69 | 3800/65 | 350/19 |
| Bandwidth (Hz/pixel) | 225 | 225 | 698 | 1445 | 1812 | 345 |
| Field of view (mm) | 200x200 | 200x200 | 256x256 | 200x200 | 368x248 | 384x384 |
| Acquisition Matrix | 320x320 | 320x320 | 256x256 | 128x128 | 184/124 | 384x384 |
| Section thickness/gap (mm) | 3/0.3 | 3/0.3 | 1.1/0 | 5/0 | 4/0.8 | 1.3/0 |
| Number of signals acquired/b-value | 3 | 2 | 1.4 | 3/b-50  4/b-300  11/b-700  Calculated b-1500 | 4/b-500  9/b-1000 | 1.4 |

^a^ *VISTA* Volumetric Isotropic TSE Acquisition = [3D fast spin echo](https://radiopaedia.org/articles/3d-fast-spin-echo-mri-sequence-1?lang=us): isotropic 3D sequences, allowing multiplanar reformats

^b^ Oblique axial = Perpendicular to the long axis of rectum of tumor

^c^ Oblique parallel = Parallel to the long axis of rectum and tumor

^d^ SPACE = Sampling perfection with application-optimized contrasts using different flip angle evolution = [3D fast spin echo](https://radiopaedia.org/articles/3d-fast-spin-echo-mri-sequence-1?lang=us): isotropic 3D sequences, allowing multiplanar reformats

**Fig. SI 1**

What is your confidence level for the tumor stage, local (Tis-T1sm2) or non-local (T1sm3-T3b)?


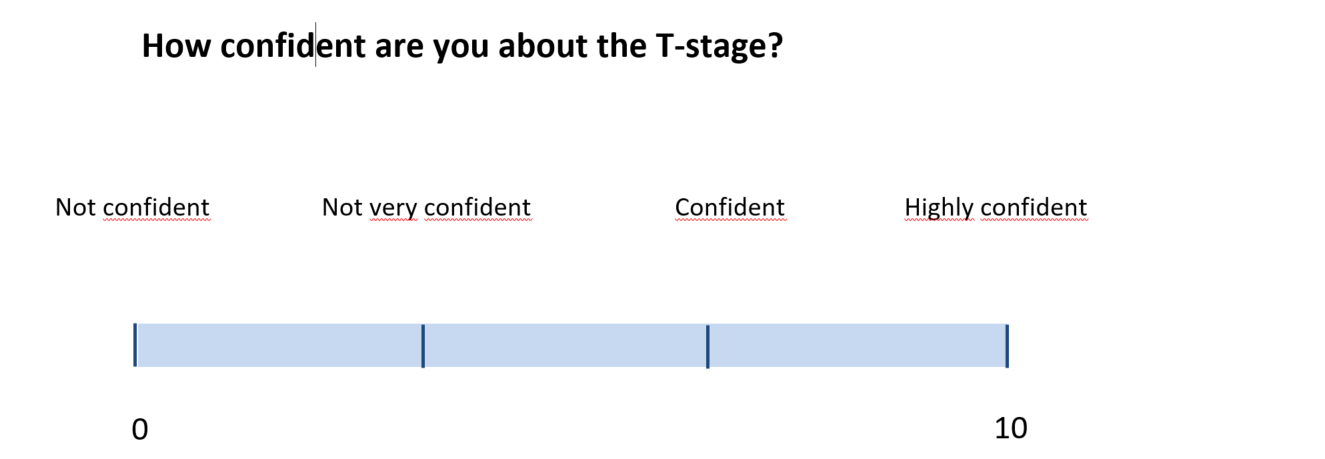


Figurative visual analog scale (fVAS) for confidence level for tumor stage with reference points at 0, 3.3, 6.6, and 10 cm

Fig. SI 2


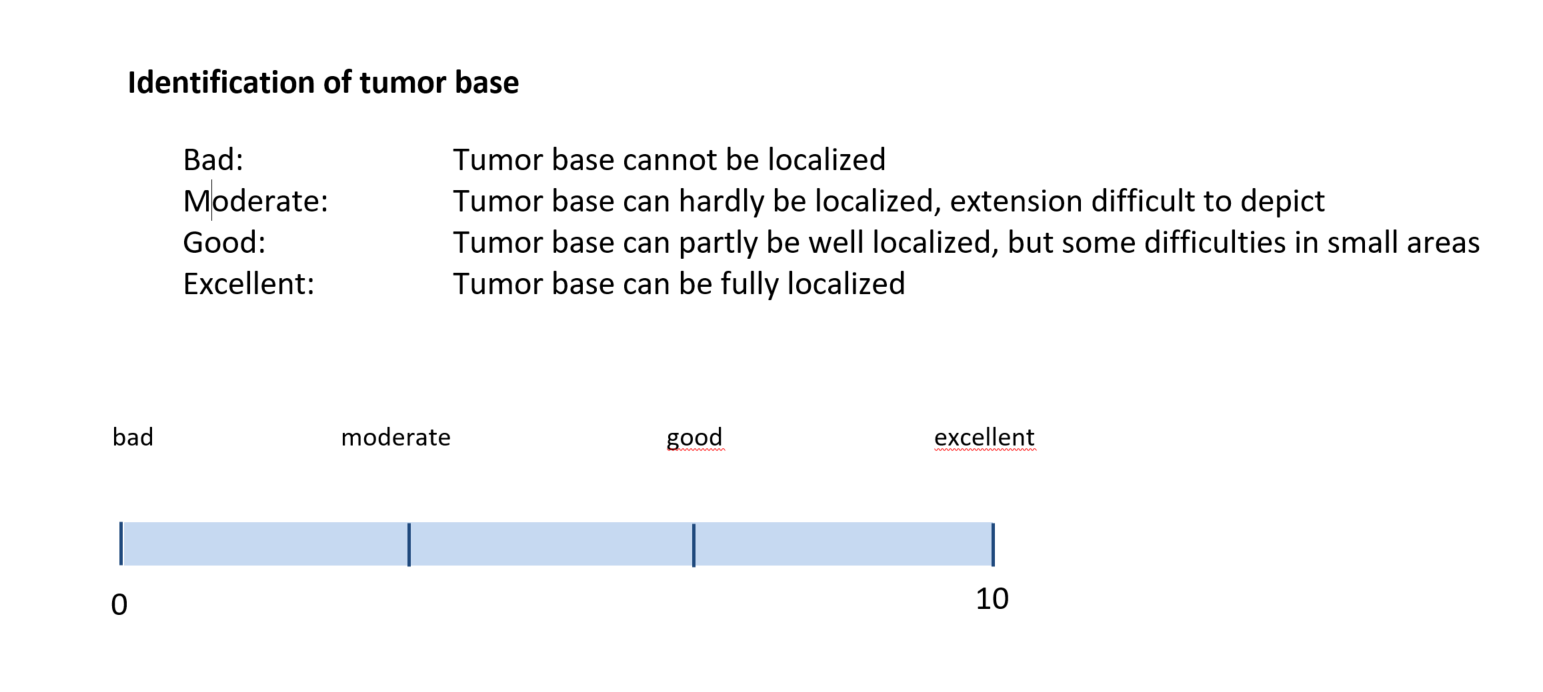


a Figurative visual analog scale (fVAS) for tumor conspicuity represented by discrimination between tumor and bowel contents and depiction of tumor base with reference points at 0, 3.3, 6.6, and 10 cm and corresponding predefined criteria for identification of tumor base.

**b Criteria for discrimination between tumor and bowel content**

Bad: No discrimination between tumor and bowel content or no luminal content, difficult to depict tumor

Moderate: Discrimination between tumor and bowel content in small areas, depiction of tumor possible, but luminal contour and form challenging to assess

Good: Good discrimination between tumor and bowel content, depiction of tumor possible, but some difficulties in assessment of luminal contour and/or form

Excellent: Excellent discrimination between tumor and bowel content, depiction of tumor, luminal tumor contour, and form

**c Criteria for delineation of tumor base**

Bad: Tumor base cannot be localized

Moderate: Tumor base can hardly be localized, extension difficult to depict

Good: Tumor base can partly be well localized, but some difficulties in small areas

Excellent: Tumor base can be fully localized

**Table SI 2** Diagnostic performance achieved by R1 and R2 using the UICC TNM8 system, without (MRex) and with (MRin) a micro-enema.

|  | **Sens % (CI), n** | **Spec % (CI), n** | **PPV % (CI)** | **NPV % (CI)** | **AUC (CI)** | **n total** |
| --- | --- | --- | --- | --- | --- | --- |
| **Reader 1 MRex** | | | | | |  |
| **Reader 1 Tis** | 91 (75 - 98), 29/32 | 78 (52 - 94), 14/18 | 88 (72 - 97) | 82 (57 - 96) | 0.84 (0.73 - 0.95) | 50 |
| **Reader 1 T1** | 17 (0 - 64), 1/6 | 91 (78 - 98), 40/44 | 20 (1-72) | 89 (76-96) | 0.54 (0.37 - 0.71) | 50 |
| **Reader 1 T2** | 11 (0 - 48), 1/9 | 98 (87 - 100), 40/41 | 50 (1 - 99) | 83 (70 - 93) | 0.54 (0.43 - 0.65) | 50 |
| **Reader 1 T3** | 100 (29 - 100), 3/3 | 85 (72 -94), 40/47 | 30 (7 - 65) | 100 (91 - 100) | 0.93 (0.87 - 0.98) | 50 |
| **Reader 1 MRin** | | | | | |  |
| **Reader 1 Tis** | 78 (60 - 91), 25/32 | 72 (47 - 90),13/18 | 83 (65 - 94) | 65 (41 - 85) | 0.75 (0.62 - 0.88) | 50 |
| **Reader 1 T1** | 0 (0 - 46), 0/6 | 84 (70 - 93), 37/44 | 0 (0 - 41) | 86 (72 - 95) | 0.42 (0.37 - 0.48) | 50 |
| **Reader 1 T2** | 44 (14 - 79), 4/9 | 98 (87 - 100), 40/41 | 80 (28 - 100) | 89 (76 - 96) | 0.71 (0.54 - 0.88) | 50 |
| **Reader 1 T3** | 100 (29 - 100), 3/3 | 89 (77 - 97), 42/47 | 38 (9 - 76) | 100 (92 - 100) | 0.95 (0.90 - 0.99) | 50 |
| **Reader 2 MRex** | | | | | |  |
| **Reader 2 Tis** | 47 (29 - 65), 15/32 | 89 (65 - 99), 16/18 | 88 (64 - 99) | 49 (31 - 67) | 0.68 (0.56 - 0.79) | 50 |
| **Reader 2 T1** | 33(4 - 24), 2/6 | 73 (57 - 85), 32/44 | 14 (2 - 43) | 89 (74 - 97) | 0.53 (0.31 - 0.75) | 50 |
| **Reader 2 T2** | 56 (21 - 86), 5/9 | 85 (71 - 94), 35/41 | 46 (17 - 77) | 90 (76 - 97) | 0.70 (0.52 - 0.89) | 50 |
| **Reader 2 T3** | 100 (29 - 100), 3/3 | 89 (77 - 97), 42/47 | 38 (9 - 76) | 100 (92 - 100) | 0.95 (92- 100) | 50 |
| **Reader 2 MRin** | | | | | |  |
| **Reader 2 Tis** | 63 (44 - 79), 20/32 | 78 (52- 94), 14/18 | 83 (63 - 95) | 54 (33 - 73) | 0.70 (0.57 - 0.83) | 50 |
| **Reader 2 T1** | 17 (0 - 64), 1/6 | 68 (52 - 81), 30/44 | 7 (0 - 32) | 86 (70 - 95) | 0.42 (0.25 - 0.60) | 50 |
| **Reader 2 T2** | 22(3- 60), 2/9 | 95 (84 - 99), 39/41 | 50 (7 - 93) | 85 (71 - 94) | 0.59 (0.44 - 0.73) | 50 |
| **Reader 2 T3** | 100 (29 - 100), 3/3 | 92 (80 - 98), 43/47 | 43 (10- 82) | 100 (92 - 100) | 0.96 (0.92 - 100) | 50 |

*Sens* sensitivity; *CI* confidence interval; *spec* specificity; *PPV* positive predictive value; *NPV* negative predictive value; *AUC* area under the curve; *n-total* total number of participants

**Table SI 3** Contingency table comparing the diagnostic performance for Reader1 and Reader2 with histopathology using the UICC TNM8 system, without (MRex) and with (MRin) a micro-enema.

| **Histopathology** | **Tis** | **T1** | **T2** | **T3** | **n-total** |
| --- | --- | --- | --- | --- | --- |
| **Reader1 MRex** | | | | | |
| **Tis** | 29 | 2 | 0 | 1 | 32 |
| **T1** | 4 | 1 | 1 | 0 | 6 |
| **T2** | 0 | 2 | 1 | 6 | 9 |
| **T3** | 0 | 0 | 0 | 3 | 3 |
| **Total** | 33 | 5 | 2 | 9 | 50 |
| **Reader1 MRin** | | | | | |
| **Tis** | 25 | 7 | 0 | 0 | 32 |
| **T1** | 5 | 0 | 1 | 0 | 6 |
| **T2** | 0 | 0 | 4 | 5 | 9 |
| **T3** | 0 | 0 | 0 | 3 | 3 |
| **Total** | 30 | 7 | 5 | 8 | 50 |
| **Reader2 MRex** | | | | | |
| **Tis** | 15 | 12 | 5 | 0 | 32 |
| **T1** | 2 | 2 | 1 | 1 | 6 |
| **T2** | 0 | 0 | 5 | 4 | 9 |
| **T3** | 0 | 0 | 0 | 3 | 3 |
| **Total** | 17 | 14 | 11 | 8 | 50 |
| **Reader2 MRin** | | | | | |
| **Tis** | 20 | 11 | 1 | 0 | 32 |
| **T1** | 4 | 1 | 1 | 0 | 6 |
| **T2** | 0 | 3 | 2 | 4 | 9 |
| **T3** | 0 | 0 | 0 | 3 | 3 |
| **Total** | 24 | 15 | 4 | 7 | 50 |
